# Sampling and coverage issues of telephone surveys used for collecting health information in Australia: results from a face-to-face survey from 1999 to 2008

**DOI:** 10.1186/1471-2288-10-77

**Published:** 2010-08-26

**Authors:** Eleonora Dal Grande, Anne W Taylor

**Affiliations:** 1 Population Research and Outcome Studies, Department of Health, South Australia, Australia; 2 Faculty of Health Sciences, The University of Adelaide, South Australia, Australia

## Abstract

**Background:**

To examine the trend of "mobile only" households, and households that have a mobile phone or landline telephone listed in the telephone directory, and to describe these groups by various socio-demographic and health indicators.

**Method:**

Representative face-to-face population health surveys of South Australians, aged 15 years and over, were conducted in 1999, 2004, 2006, 2007 and 2008 (n = 14285, response rates = 51.9% to 70.6%). Self-reported information on mobile phone ownership and usage (1999 to 2008) and listings in White Pages telephone directory (2006 to 2008), and landline telephone connection and listings in the White Pages (1999 to 2008), was provided by participants. Additional information was collected on self-reported health conditions and health-related risk behaviours.

**Results:**

Mobile only households have been steadily increasing from 1.4% in 1999 to 8.7% in 2008. In terms of sampling frame for telephone surveys, 68.7% of South Australian households in 2008 had at least a mobile phone or landline telephone listed in the White Pages (73.8% in 2006; 71.5% in 2007). The proportion of mobile only households was highest among young people, unemployed, people who were separated, divorced or never married, low income households, low SES areas, rural areas, current smokers, current asthma or people in the normal weight range. The proportion with landlines or mobiles telephone numbers listed in the White Pages telephone directory was highest among older people, married or in a defacto relationship or widowed, low SES areas, rural areas, people classified as overweight, or those diagnosed with arthritis or osteoporosis.

**Conclusion:**

The rate of mobile only households has been increasing in Australia and is following worldwide trends, but has not reached the high levels seen internationally (12% to 52%). In general, the impact of mobile telephones on current sampling frames (exclusion or non-listing of mobile only households or not listed in the White Pages directory) may have a low impact on health estimates obtained using telephone surveys. However, researchers need to be aware that mobile only households are distinctly different to households with a landline connection, and the increase in the number of mobile-only households is not uniform across all groups in the community. Listing in the White Pages directory continues to decrease and only a small proportion of mobile only households are listed. Researchers need to be aware of these telephone sampling issues when considering telephone surveys.

## Background

With the rapid changes to the telecommunications industry, it is unknown whether telephone surveys can continue to be used to reliably collect representative information regarding health status and health risk behaviours. Telephone surveys, traditionally using landline telephones, have been used to collect and monitor health-related information over the last 30 years [1-3] and have been used to determine the prevalence of chronic conditions, health-related risk behaviours, and assess knowledge, attitudes, and opinions on health issues. Telephone surveys using Computer Assisted Telephone Interviewing (CATI) technology have been seen as a cost-effective and timely method of collecting health information [4-9] and have provided greater standardisation of administration through closer supervision of interviewers compared to traditional face-to-face methods.

In Australia, for the last decade, the coverage of households with a telephone connected (landline) and the adequacy of the sampling frame(s) have been a concern for those involved in epidemiologically-sound telephone surveys. The proportion of people who do not have a landline telephone connected in the household is not uniformly distributed in the population[10,11] and there is difficulty in obtaining a complete sampling frame[12]. With the exception of the Northern Territory, the landline telephone coverage in Australian households has been historically very high (96.8% in South Australia in 2002[10], 94.4% in Australia in 1991[13]). There are two main population sampling methods used in Australia: Australian Electronic White Pages (EWP) directory and Random Digit Dialling (RDD). EWP consists of all landline telephone numbers, names and address details for a household or business. All these telephone numbers are centrally located and routinely listed in the EWP regardless of the telecommunication carrier. Households can opt, at a cost, to not have their telephone number have listed in the EWP (also known as silent numbers). Mobile numbers are not routinely listed in the EWP, so owners can choose, at a cost, to have their mobile number listed in the EWP. This exclusion of unlisted numbers from the sampling frame can have an effect on the estimates for the population in telephone surveys and remains an important concern for researchers [11,12]. RDD methods in Australia are based on the prefixes of the telephone numbers in the EWP to generate a sampling frame in order to include the silent numbers. This method is referred to as list-assisted RDD (LA-RDD). However, these RDD sampling methods do not include mobile numbers in their sampling frame. The differences and similarities between these two methods, in the Australian context, have been described elsewhere [10-12,14].

Since the early 2000 s, international trends have seen households change to new telecommunications technologies, whereby individuals in the household are solely contactable by mobile phones or other means such as Voice over Internet Protocol (VoIP), not by the traditional landline telephone. This has negatively impacted on telephone surveys and sampling methodologies. International studies have shown a dramatic increase in mobile only households (those not having a landline telephone connected to the household). In 2006, 11.8% of households in the United States [15], 52% in Finland [16] and 17% in France [16] were mobile only. In 2002, 13.1% of households in Italy were mobile only households [17].

As a result of these rapid changes both in Australia and worldwide, determining an adequate sampling frame to include these non-traditional telephone numbers and to demarcate geographic locations, is becoming increasingly important. This paper presents how two factors impact household telephone surveys in Australia; the presence of mobile only individuals and the lack of full enumeration of telephone numbers in a telephone directory. Mobile only households are not covered in the RDD sampling frame and unlisted telephone numbers (landlines and mobiles) are not covered in the EWP telephone directory. Both samplings frames exclude people with no mobiles or landline telephones. The aim of this study is to examine the trend of mobile only households and households that have a mobile telephone or landline telephone listing in the EWP, and to describe these groups by various socio-demographics and health indicators to determine the potential bias due to non-coverage in the sample in the Australian context.

## Methods

### Survey design and sample section

Questions regarding landline telephone, mobile, and internet connections were included in the 1999, 2004, 2006, 2007 and 2008 South Australian Health Omnibus Surveys (HOS)[18,19]. HOS is a multi-stage, systematic, clustered area sample of households conducted face-to-face annually in spring based on the Australian Bureau of Statistics (ABS) collector districts (CDs). The HOS samples included households randomly selected from CDs, from the metropolitan Adelaide area and country towns with a population of 1,000 people or more. Within each CD, a random starting point was selected and from this point 10 households were then selected in a given direction with a fixed skip interval. Hotels, motels, hospitals, hostels and other institutions were excluded from the sample. Approach letters were sent to selected households informing them of the survey. One person aged 15 years or over, who was last to have a birthday, was randomly selected from each household for interview. The interviews were conducted in people's homes by trained interviewers. Up to six call back visits were made to the chosen households to interview the selected person. There was no replacement for non-respondents. Response rates (and sample size) from the surveys were 70.6% (n = 3012) in 1999, 68.4% (n = 2982) in 2004, 54.9% (n = 2969) in 2006, 51.9% (n = 2507) in 2007 and 53.6% (n = 2824) in 2008. Each individual data set was weighted by five year age groups, sex, area (metropolitan Adelaide and SA country) and household size to the most recent ABS Census or Estimated Residential Population for South Australia to provide population estimates.

### Data items

Questions about landline telephone connection and listing in the EWP (alphabetic directory of non-silent phone numbers belonging to residential households and businesses which include surname and address details) were included in the 1999, 2004, 2006, 2007 and 2008 surveys. These surveys also included questions on mobile phone ownership and usage in the household. Additional questions on mobile phone listing in the EWP, and future landline and mobile phone ownership plans were included in 2006, 2007 and 2008.

Identical demographic variables included in each survey were age, sex, area of residence, country of birth, education level, marital status, gross annual household income, and work status. The Index of Relative Social Disadvantage (IRSD) developed by the ABS was also calculated to identify the geographical areas that were relatively disadvantaged[20]. The IRSD is a composite measure based on selected Census variables such as income, educational attainment and employment status. The IRSD scores were grouped into quintiles for analysis where the highest quintile comprises postcodes with the highest IRSD scores (most advantaged areas).

Chronic conditions included medically confirmed diabetes, current asthma, arthritis and osteoporosis. Self-reported health risk factor data included smoking status and body mass index (BMI) which was derived from self-reported weight and height and recoded into four categories (underweight, normal weight, overweight and obese) [21]. Mobile only households were defined as households with no landline telephone connected to the house and if mobile phones were currently being used by members of the household.

The questionnaire and methodology for these surveys were approved by the Human Research Ethics Committee of the South Australian Department of Health (SA Health).

### Data analyses

Data analysis was conducted using SPSS for Windows Version 15.0. The conventional p value of 0.05 was used as the criterion for statistical significance. To compare prevalence over time (from 1999 to 2008), χ^2 ^test for trend was used for mobile only households, households with landline telephone only connections and households with both a landline telephone connection and mobile. A comparison between 2006, 2007 and 2008 using χ^2 ^tests for trend was undertaken to assess for change in various socio-demographic and health-related variables between mobile only households and households with a landline or a mobile telephone number listed in the EWP.

Separate univariate analyses using χ^2 ^tests were undertaken for 2006, 2007 and 2008, to assess mobile only households, and households with a landline or a mobile telephone number listed in the EWP on a range of socio-demographic and health-related variables.

## Results

Table 1 shows the trends of mobile only and landline households from 1999, 2004, 2006 to 2008. The proportion of mobile only households has been steadily increasing over the eight years (χ^2^_trend _= 177.01, p < 0.001). There was a statistically significant decline in the proportion of households with landline telephone only connections (χ^2^_trend _= 1693.6, p < 0.001) from 44.6% in 1999 to 6.9% in 2008, and a statistically significant increase in households with both a landline telephone connection and mobile (χ^2^_trend _= 1188.6, p < 0.001) from 52.7% in 1999 to 83.8% in 2008.

**Table 1:** Telephone (landline) and mobile status of household, and household has landline and/or mobile telephone listed in the Australian Electronic White Pages by year

		Telephone (landline) and mobile status of household						Household has landline and/or at least one mobile telephone listed in directory	
		No landline telephone or mobile	Mobile only household	Landline telephone only	Landline telephone and mobile	Not stated		No	Yes
Year	n	% (95% CI)	% (95% CI)	% (95% CI)	% (95% CI)	%	n	% (95% CI)	% (95% CI)
1999	3012	1.3 (1.0 - 1.8)	1.4 (1.0 - 1.8)	44.6 (42.9 - 46.4)	52.7 (50.9 - 54.5)				
2004	2982	1.2 (0.8 - 1.6)	3.4 (2.8 - 4.1)	95.4 (94.6 - 96.1) *		0.1			
2006	2969	0.3 (0.1 - 0.5)	5.2 (4.4 - 6.0)	9.7 (8.7 - 10.8)	84.7 (83.4 - 86.0)	0.1	2961	26.2 (24.6 - 27.8)	73.8 (72.2 - 75.4)
2007	2507	1.1 (0.7 - 1.6)	7.1 (6.1 - 8.1)	11.1 (9.9 - 12.3)	80.6 (79.0 - 82.1)	0.2	2480	28.5 (26.8 - 30.3)	71.5 (69.7 - 73.2)
2008	2824	0.3 (0.2 - 0.6)	8.7 (7.7 - 9.8)	6.9 (6.1 - 7.9)	83.8 (82.4 - 85.1)	0.3	2814	31.3 (29.5 - 32.9)	68.7 (66.7 - 70.1)

* In 2004 HOS, households with a landline were not asked if there were any person in the household that had a mobile phone

Respondents were asked if their mobile or landline telephones were listed in the EWP. Of mobile only households, 6.9% had their mobile number listed in the EWP in 2008 (8.0% in 2006; 3.4% in 2007). Of households with both a mobile and landline telephone connected, 7.4% had their mobile number listed in 2008 (7.3% in both 2006 and 2007). Examination of households with a landline telephone connection revealed that 77.0% of these households in 2006, 76.3% in 2007 and 74.1% in 2008, had their landline telephone numbers listed in the EWP (χ^2^_trend _= 18.6, p < 0.001). Hence, 68.7% of South Australian households in 2008 had at least a mobile phone and/or landline telephone listed in the EWP (73.8% in 2006, 71.5% in 2007) (Table 1).

When the proportion of mobile only household respondents was compared on selected demographics and health indicators over the three years (2006 to 2008) (Table 2), increased trends were significant for a wide range of variables. When households with a mobile or landline telephone number listed in the EWP (Table 3) were examined over the three years (2006 to 2008), decreasing trends were apparent for a range of variables.

**Table 2:** Proportion of people living in mobile only households by selected demographic, health conditions, and health related risk factors from 2006 to 2008

		2006			2007			2008		Test for trend
	n	% (95% CI)	**P value**^**1**^	n	% (95% CI)	**P value**^**1**^	n	% (95% CI)	**P value**^**1**^	**P value**^**2**^
**DEMOGRAPHICS**										
**Gender**			**0.007**			**< 0.001**			0.149	
Male	92/1460	6.3 (5.2 - 7.7)		109/1222	8.9 (7.4 - 10.6)		131/1382	9.4 (8.0 - 11.1)		**0.002**
Female	62/1509	4.1 (3.2 - 5.2)		68/1285	5.3 (4.2 - 6.7)		114/1433	7.9 (6.6 - 9.4)		**< 0.001**
	
**Age Groups**			**< 0.001**			**< 0.001**			**< 0.001**	
15 to 29 years	73/708	10.4 (8.3 - 12.8)		73/587	12.7 (10.2 - 15.7)		114/571	17.9 (15.2 - 20.9)		**< 0.001**
30 to 44 years	47/777	6.1 (4.7 - 8.1)		67/644	10.5 (8.4 - 13.2)		76/693	11.0 (8.9 - 13.6)		**0.001**
45 years and over	34/1485	2.3 (1.6 - 3.2)		38/1276	3.0 (2.2 - 4.0)		43/1420	3.0 (2.2 - 4.0)		0.213
	
**Country of Birth**			0.935			**0.001**			**< 0.001**	
Australia	116/2197	5.3 (4.4 - 6.3)		152/1911	8.0 (6.8 - 9.3)		209/2110	9.9 (8.7 - 11.3)		**< 0.001**
UK & Ireland	17/354	4.9 (3.1 - 7.7)		5/242	1.9 #		6/296	2.1 (1.0 - 4.5)		**0.040**
Other	21/418	5.0 (3.3 - 7.5)		20/354	5.8 (3.8 - 8.7)		29/409	7.0 (4.9 - 9.8)		0.218
	
**Marital status **^**a**^			**< 0.001**			**< 0.001**			**< 0.001**	
Married/defacto	66/1862	3.5 (2.8 - 4.5)		53/1534	3.5 (2.7 - 4.5)		111/1765	6.3 (5.2 - 7.5)		**< 0.001**
Separated/divorced	24/248	9.6 (6.5 - 13.9)		36/227	16.0 (11.8 - 21.3)		27/237	11.3 (7.8 - 15.9)		0.560
Widowed	3/156	1.6 #		4/141	3.1 #		5/149	3.6 (1.6 - 7.9)		0.286
Never married	62/694	8.9 (7.0 - 11.2)		83/602	13.8 (11.3 - 16.8)		101/661	15.2 (12.6 - 18.1)		**< 0.001**
	
**Educational Attainment **^**b**^			**0.007**			**< 0.001**			**0.002**	
None to secondary schooling	81/1385	5.8 (4.7 - 7.2)		107/1234	8.7 (7.2 - 10.4)		134/1281	10.4 (8.9 - 12.2)		**< 0.001**
Trade qualifications, Certificate, Diploma	64/1086	5.8 (4.6 - 7.4)		60/843	7.1 (5.6 - 9.1)		82/1015	8.1 (6.6 - 9.9)		**0.042**
Bachelor Degree or higher	10/488	2.1 (1.1 - 3.8)		10/428	2.4 (1.3 - 4.3)		27/514	5.2 (3.6 - 7.5)		**0.005**

**Area of residence**			0.089			**< 0.001**			**< 0.001**	
Metropolitan	101/2123	4.8 (3.9 - 5.8)		107/1847	5.8 (4.8 - 7.0)		160/2152	7.4 (6.4 - 8.6)		**< 0.001**
Country	53/846	6.3 (4.8 - 8.1)		70/660	10.6 (8.5 - 13.2)		84/663	12.6 (10.3 - 15.4)		**< 0.001**
	
**Annual household income**			**0.001**			**0.001**			**0.001**	
Up to $20,000	25/387	6.5 (4.4 - 9.4)		38/335	11.2 (8.3 - 15.1)		34/342	9.8 (7.1 - 13.4)		0.105
$20,001-$40,000	42/509	8.2 (6.1 - 10.9)		33/437	7.6 (5.5 - 10.5)		44/431	10.2 (7.7 - 13.4)		0.297
$40,001-$60,000	24/451	5.3 (3.6 - 7.8)		28/383	7.2 (5.0 - 10.2)		41/353	11.7 (8.8 - 15.5)		**< 0.001**
$60,001-$80,000	11/350	3.1 (1.7 - 5.4)		22/274	8.0 (5.3 - 11.8)		23/326	7.0 (4.7 - 10.3)		**0.027**
$80,001 or more	20/675	2.9 (1.9 - 4.5)		22/600	3.7 (2.5 - 5.5)		42/784	5.4 (4.0 - 7.2)		**0.018**
Not stated	33/597	5.6 (4.0 - 7.7)		34/477	7.2 (5.2 - 9.9)		61/580	10.4 (8.2 - 13.1)		**0.002**
	
**Work status **^**c**^			**< 0.001**			**< 0.001**			**< 0.001**	
Work full time	69/1109	6.2 (4.9 - 7.8)		76/920	8.2 (6.6 - 10.2)		109/1091	10.0 (8.4 - 11.9)		**0.001**
Work part time	30/566	5.2 (3.7 - 7.4)		27/455	5.9 (4.1 - 8.5)		40/494	8.2 (6.1 - 10.9)		0.055
Home Duties	16/272	5.7 (3.5 - 9.2)		28/255	10.9 (7.7 - 15.4)		26/227	11.6 (8.1 - 16.5)		**0.019**
Unemployed	16/79	20.9 (13.4 - 31.1)		22/76	28.6 (19.7 - 39.6)		19/77	24.1 (15.9 - 34.8)		0.634
Retired	3/578	0.6 #		6/505	1.2 (0.6 - 2.6)		10/575	1.8 (1.0 - 3.2)		0.068
Student	9/220	4.1 (2.2 - 7.6)		9/217	4.2 (2.2 - 7.7)		23/227	10.0 (6.7 - 14.6)		**0.009**
Other/Not working because of work related injury	11/104	10.8 (6.2 - 18.2)		10/79	12.3 (6.8 - 21.3)		15/123	12.3 (7.6 - 19.3)		0.735
	
**SEIFA IRSD Quintiles **^**d**^			**0.001**			**< 0.001**			**< 0.001**	
Lowest/low (most disadvantaged)	90/1293	7.0 (5.7 - 8.5)		118/1124	10.5 (8.9 - 12.4)		159/1247	12.7 (11.0 - 14.7)		**< 0.001**
Middle	25/578	4.3 (2.9 - 6.3)		31/496	6.3 (4.5 - 8.8)		31/520	5.9 (4.2 - 8.3)		0.314
High/Highest (least disadvantaged)	39/1088	3.6 (2.6 - 4.9)		25/882	2.8 (1.9 - 4.1)		55/1048	5.3 (4.1 - 6.8)		**0.017**

**HEALTH CONDITIONS** **AND HEALTH RELATED** **RISK FACTORS**										
**Diabetes**	13/197	6.4 (3.8 - 10.8)	0.413	6/168	3.3 (1.5 - 7.3)	0.051	24/214	11.0 (7.5 - 15.9)	0.181	0.053
**Arthritis**	18/694	2.6 (1.6 - 4.0)	**< 0.001**	27/595	4.6 (3.2 - 6.5)	**0.006**	31/707	4.3 (3.1 - 6.1)	**< 0.001**	0.092
**Osteoporosis **	2/184	1.3 #	**0.013**	6/168	3.3 (1.5 - 7.3)	**0.051**	3/158	2.1 #	**0.002**	0.618
**Asthma (current)**	37/364	10.1 (7.4 - 13.6)	**< 0.001**	31/290	10.7 (7.7 - 14.8)	**0.009**	47/385	12.1 (9.2 - 15.8)	**0.009**	0.377
	
**Smoking status **^**e**^			**< 0.001**			**< 0.001**			**< 0.001**	
Non/Ex smoker	78/2357	3.3 (2.7 - 4.1)		87/2009	4.3 (3.5 - 5.3)		138/2264	6.1 (5.2 - 7.2)		**< 0.001**
Current smoker	76/611	12.4 (10.1 - 15.3)		90/495	18.2 (15.1 - 21.9)		106/551	19.2 (16.2 - 22.7)		**0.002**
	
**Body mass index (BMI) **^**f**^			0.058			**< 0.001**			**0.049**	
Underweight <18.5	3/64	4.8 #		14/76	18.9 (11.7 - 29.1)		4/63	5.7 #		0.998
Normal 18.5-24.9	65/1144	5.7 (4.5 - 7.2)		79/925	8.5 (6.9 - 10.5)		99/1005	9.8 (8.2 - 11.8)		**< 0.001**
Overweight 25.0-29.9	28/848	3.3 (2.3 - 4.7)		47/769	6.1 (4.6 - 8.0)		52/820	6.4 (4.9 - 8.2)		**0.005**
Obese 30.0+	33/565	5.9 (4.2 - 8.2)		20/471	4.2 (2.7 - 6.4)		46/560	8.3 (6.3 - 10.8)		0.081

**Overall**	154/2969	5.2 (4.5 - 6.1)		177/2507	7.1 (6.1 - 8.1)		244/2816	8.7 (7.7 - 9.8)		**< 0.001**

1 p values that are bold denotes statistical significance at the 0.05 level from the X^2 ^test for that variable;

2 p values that are bold denotes statistical significance at the 0.05 level from the X^2 ^test for trend for the 2006 to 2008 time period for that category; CI confidence interval of proportion

^**a **^10 cases missing for 2006, 4 cases missing for 2007 and 7 cases missing for 2008; ^**b **^9 cases missing for 2006, 1 case missing for 2007 and 9 cases missing for 2008; ^**c **^42 cases missing for 2006, 1 case missing for 2007 and 10 cases missing for 2008; ^**d **^10 cases missing for 2006, 5 cases missing for 2007; ^**e **^2 cases missing for 2006, 2 cases missing for 2007; ^**f **^348 cases missing for 2006, 265 cases missing for 2007 and 369 cases for 2008

**Table 3:** Proportion of people living in households where mobile phone or landline telephone is listed in the White Pages by selected demographic, health conditions, and health related risk factors from 2006 to 2008

		2006			2007			2008		Test for trend
	n	% (95% CI)	**P value**^**1**^	n	% (95% CI)	**P value**^**1**^	n	% (95% CI)	**P value**^**1**^	**P value**^**2**^
**DEMOGRAPHICS**										
**Gender**			0.625			0.557			0.842	
Male	1106/1507	74.2 (71.9 - 76.4)		915/1271	70.9 (68.3 - 73.4)		989/1436	68.5 (66.0 - 70.9)		**0.006**
Female	1079/1454	73.4 (71.1 - 75.6)		857/1209	72.0 (69.5 - 74.4)		944/1379	68.9 (66.4 - 71.2)		**0.001**
	
**Age Groups**			**< 0.001**			**< 0.001**			**< 0.001**	
15 to 29 years	440/707	62.2 (58.6 - 65.7)		317/571	55.6 (51.5 - 59.6)		306/570	53.3 (49.6 - 57.0)		**0.002**
30 to 44 years	554/773	71.7 (68.4 - 74.7)		429/636	67.4 (63.6 - 70.9)		438/692	63.3 (59.6 - 66.8)		**0.001**
45 to 59 years	563/751	75.0 (71.8 - 78.0)		466/627	74.4 (70.9 - 77.7)		493/676	73.0 (69.5 - 76.2)		0.384
60 years and over	628/730	86.1 (83.4 - 88.4)		560/647	86.7 (83.8 - 89.1)		628/746	84.3 (81.5 - 86.7)		0.313
	
**Country of Birth**			0.639			**0.004**			**< 0.001**	
Australia	1627/2191	74.3 (72.4 - 76.1)		1367/1886	72.5 (70.4 - 74.4)		1460/2104	69.4 (67.4 - 71.3)		**< 0.001**
UK & Ireland	256/353	72.7 (67.8 - 77.0)		179/241	74.4 (68.5 - 79.5)		221/298	74.3 (69.0 - 78.9)		0.633
Other	302/417	72.4 (68.0 - 76.5)		227/353	64.2 (59.0 - 69.0)		251/413	60.9 (56.2 - 65.5)		**< 0.001**
	
**Marital status **^**a**^			**< 0.001**			**< 0.001**			**< 0.001**	
Married/defacto	1465/1860	78.8 (76.8 - 80.5)		1188/1525	77.9 (75.8 - 79.9)		1301/1764	73.8 (71.7 - 75.8)		**< 0.001**
Separated/divorced	155/246	62.9 (56.7 - 68.7)		138/222	61.9 (55.3 - 68.0)		142/236	60.0 (53.7 - 66.1)		0.518
Widowed	118/154	76.5 (69.2 - 82.5)		110/141	78.2 (70.7 - 84.2)		118/148	79.9 (72.7 - 85.5)		0.483
Never married	444/690	64.4 (60.8 - 67.9)		336/588	57.1 (53.1 - 61.0)		370/659	56.1 (52.3 - 59.9)		**0.002**
	
**Educational Attainment **^**b**^			**0.032**			0.494			**< 0.001**	
None to secondary schooling	990/1378	71.8 (69.4 - 74.1)		854/1216	70.2 (67.6 - 72.7)		840/1275	65.9 (63.2 - 68.4)		**0.001**
Trade qualifications, Certificate, Diploma	826/1085	76.1 (73.4 - 78.5)		611/836	73.1 (70.0 - 76.0)		741/1016	73.0 (70.2 - 75.6)		0.105
Bachelor Degree or higher	361/488	74.0 (70.0 - 77.7)		307/427	72.0 (67.5 - 76.0)		348/514	67.7 (63.6 - 71.6)		**0.027**

**Area of residence**			**< 0.001**			**< 0.001**			**0.001**	
Metropolitan	1523/2120	71.8 (69.9 - 73.7)		1264/1830	69.1 (66.9 - 71.2)		1443/2152	67.1 (65.0 - 69.0)		**0.001**
Country	663/841	78.8 (75.9 - 81.5)		508/650	78.2 (74.9 - 81.2)		490/663	74.0 (70.5 - 77.2)		**0.030**
	
**Annual household** **income**			0.121			**0.005**			**0.039**	
Up to $20,000	486/675	74.5 (69.9 - 78.6)		446/597	66.2 (60.9 - 71.1)		569/784	64.1 (58.9 - 69.1)		0.812
$20,001-$40,000	273/350	74.5 (70.6 - 78.1)		206/273	74.3 (70.0 - 78.2)		229/328	68.7 (64.1 - 72.9)		**0.013**
$40,001-$60,000	341/449	75.9 (71.8 - 79.6)		269/380	70.8 (66.0 - 75.1)		241/353	68.3 (63.3 - 72.9)		**0.016**
$60,001-$80,000	379/509	78.1 (73.5 - 82.2)		322/434	75.6 (70.1 - 80.3)		295/429	69.8 (64.7 - 74.5)		**0.049**
$80,001 or more	284/381	72.0 (68.5 - 75.2)		218/330	74.6 (71.0 - 78.0)		216/336	72.6 (69.4 - 75.6)		**0.003**
Not stated	422/597	70.8 (67.0 - 74.3)		312/467	66.7 (62.3 - 70.8)		384/585	65.6 (61.7 - 69.3)		0.058
	
**Work status **^**c**^			**< 0.001**			**< 0.001**			**< 0.001**	
Work full time	796/1109	71.8 (69.1 - 74.4)		641/909	70.4 (67.4 - 73.3)		751/1091	68.9 (66.0 - 71.5)		0.127
Work part time	422/565	74.7 (71.0 - 78.2)		327/450	72.7 (68.4 - 76.6)		346/494	70.0 (65.8 - 73.9)		0.084
Home Duties	198/270	73.2 (67.7 - 78.2)		170/252	67.2 (61.2 - 72.7)		134/222	60.2 (53.7 - 66.4)		**0.002**
Unemployed	39/78	50.3 (39.4 - 61.1)		35/74	47.8 (36.8 - 59.0)		36/76	47.2 (36.4 - 58.3)		0.707
Retired	489/574	85.2 (82.1 - 87.9)		435/504	86.2 (82.9 - 88.9)		482/575	83.8 (80.6 - 86.6)		0.502
Student	141/220	64.3 (57.8 - 70.3)		129/215	59.8 (53.2 - 66.2)		114/226	50.5 (44.1 - 57.0)		**0.003**
Other/Not working because of work related injury	71/104	68.1 (58.6 - 76.3)		35/74	48.1 (37.1 - 59.3)		67/121	55.4 (46.5 - 64.0)		0.067
	
**SEIFA IRSD** **Quintiles **^**d**^			**0.080**			**< 0.001**			**< 0.001**	
Lowest/low (most disadvantaged)	827/1088	72.0 (69.5 - 74.4)		666/876	67.5 (64.7 - 70.2)		804/1237	64.9 (62.2 - 67.5)		**0.001**
Middle	421/575	73.3 (69.5 - 76.7)		359/495	72.5 (68.4 - 76.2)		368/520	70.7 (66.7 - 74.5)		0.634
High/Highest (least disadvantaged)	927/1288	76.0 (73.4 - 78.5)		745/1103	76.0 (73.1 - 78.7)		761/1048	72.6 (69.9 - 75.2)		0.123

**HEALTH CONDITIONS** **AND HEALTH RELATED** **RISK FACTORS**										
**Diabetes**	144/197	73.3 (66.7 - 79.0)	0.863	132/168	78.7 (71.9 - 84.2)	**0.033**	158/214	73.9 (67.7 - 79.4)	0.104	0.994
**Arthritis**	550/693	79.4 (76.2 - 82.2)	**< 0.001**	452/593	76.2 (72.6 - 79.5)	**0.003**	534/705	75.8 (72.5 - 78.8)	**< 0.001**	0.090
**Osteoporosis **	161/184	87.4 (81.9 - 91.5)	**< 0.001**	132/168	78.7 (71.9 - 84.2)	**0.033**	111/158	70.3 (62.8 - 76.9)	0.703	**< 0.001**
**Asthma (current)**	256/364	70.3 (65.4 - 74.8)	0.105	193/283	68.3 (62.6 - 73.4)	0.203	245/385	63.6 (58.7 - 68.3)	**0.017**	**0.048**
	
**Smoking status **^**e**^			**< 0.001**			**< 0.001**			**< 0.001**	
Non/Ex smoker	1817/2351	77.3 (75.5 - 78.9)		1492/1994	74.9 (72.9 - 76.7)		1642/2262	72.6 (70.7 - 74.4)		**< 0.001**
Current smoker	368/608	60.5 (56.6 - 64.3)		280/486	57.6 (53.2 - 61.9)		291/544	53.6 (49.4 - 57.7)		**0.014**
	
**Body mass index** **(BMI) **^**f**^			**0.011**			**< 0.001**			**< 0.001**	
Underweight <18.5	44/64	68.9 (56.8 - 78.9)		36/76	47.7 (36.9 - 58.7)		43/63	67.2 (54.9 - 77.5)		0.963
Normal 18.5-24.9	816/1140	71.5 (68.8 - 74.1)		635/923	68.7 (65.7 - 71.6)		641/1005	63.8 (60.8 - 66.7)		**< 0.001**
Overweight 25.0-29.9	657/847	77.6 (74.7 - 80.3)		569/762	74.7 (71.5 - 77.6)		602/820	73.5 (70.4 - 76.4)		**0.053**
Obese 30.0+	428/565	75.8 (72.1 - 79.2)		350/463	75.6 (71.5 - 79.3)		402/560	71.7 (67.9 - 75.3)		0.108

**Overall**	2186/2961	73.8 (72.2 - 75.4)		1773/2480	71.5 (69.7 - 73.2)		1993/2814	68.7 (66.9 - 70.4)		**< 0.001**

1 p values that are bold denotes statistical significance at the 0.05 level from the X^2 ^test for that variable; 2 p values that are bold denotes statistical significance at the 0.05 level from the X^2 ^test for trend for the 2006 to 2008 time period for that category; CI confidence interval of proportion

^**a **^10 cases missing for 2006, 4 cases missing for 2007 and 7 cases missing for 2008; ^**b **^9 cases missing for 2006, 1 case missing for 2007 and 9 cases missing for 2008; ^**c **^42 cases missing for 2006, 1 case missing for 2007 and 10 cases missing for 2008; ^**d **^10 cases missing for 2006, 5 cases missing for 2007; ^**e **^2 cases missing for 2006; ^**f **^345 cases missing for 2006, 265 cases missing for 2007 and 366 cases for 2008

When examined by selected demographics for 2006, 2007 and 2008 (Table 2), respondents who lived in a mobile only household were statistically significantly more likely to be in the younger age groups, separated, divorced or never married, unemployed, born in Australia, have at least obtained secondary schooling, living in rural areas of South Australia, from low income households or from low SES areas of South Australia, and statistically significantly less likely to be in the older age groups, widowed, married or in a defacto relationship, born in UK/Ireland, living in metropolitan Adelaide, from high income households ($80,000 or more per annum), retired, to have a bachelor degree or higher and from high SES areas of South Australia. In terms of health conditions and health related risk factors, respondents who live in mobile only households were statistically significantly more likely to be current smokers, classified as having normal BMI or diagnosed with current asthma, and were statistically significantly less likely to be classified as overweight.

Respondents from households with a mobile or landline telephone number listed in the EWP (Table 3) were statistically significantly more likely to be in the older age groups, married or in a defacto relationship or widowed, retired, living in rural areas and from high SES areas of South Australia, and statistically significantly less likely to be in the younger age groups, never married, separated or divorced, a student, unemployed or not working because of an injury, living in metropolitan Adelaide or from low SES areas of South Australia. They were also statistically significantly more likely to be classified as being overweight, to have arthritis, or statistically significantly less likely to be current smokers and have normal BMI.

Further questions were asked to determine the likelihood of people with a landline telephone switching to being a mobile only household. Overall, 6.9% in 2006, 5.9% in 2007, and 8.1% in 2008 indicated that they were 'very likely' while 11.0%, 10.8%, and 12.1% respectively indicated they were 'somewhat likely' to discontinue their landline connection.

## Discussion

This study has shown, using large representative surveys, the proportion of mobile only households has been increasing in Australia and is following international trends. The prevalence of mobile only households in South Australia among people aged 15 years and over (8.7% in 2008), is not as high as other international studies: 11.8% in the United States[15]; 52% in Finland[16] and 17% in France in 2006[16] and, 13.1% in Italy in 2002[17]. However, the pattern of increasing prevalence remains the same and there are also changes among a range of demographic, health status and health risk behaviours groups. The prevalence of households with neither a mobile phone nor landline telephone has remained low and is likely to have a minimal effect on surveys using mobile phone or landline telephones. However, the mobile only prevalence may increase in South Australia over the next few years since 8% of survey respondents indicated they were very likely to become a mobile only household.

From this study, using LA-RDD methodology to generate a sampling frame to include unlisted landline telephone numbers excludes mobile only households as well as households with no landline telephone connection which is 9% of the population. This could be considered small [22] and one could argue that excluding this group would have minimal impact on health estimates. However, the results presented in this study indicate that mobile only households have different demographic characteristics to households with landline and/or mobiles. These demographic differences are similar to US studies [15,23] with a higher proportion of males, younger people, people who are unemployed, separated, divorced or never married, people living in rural areas of South Australia, and low SES households (low income households and reside in the most disadvantaged areas) living in mobile only households. From this study, in terms of health indicators, people classified as overweight, having current asthma and current smokers would also be under-represented in these surveys.

There are some data quality and collection issues that need to be taken into account when including mobile telephones into the sample frame. One is the location or the situation of the respondent at the time of the interview: respondents may choose not to answer a call to save battery life; answering a call which may incur a cost to both the respondent and the researcher (if the respondent is overseas the fee may be much higher depending on distance from Australia and contractual agreement with individual telecommunication providers); and safety and legal issues, eg the respondent may be driving and using their mobile (texting and talking) at the same time which is illegal in Australia [16]. A study conducted in the US [24] found that those respondents who participated in the survey using a mobile phone, 56% were at home while undertaking the survey, 14% were driving and 13% were at work. The remaining respondents were at other locations such as in public areas, in another person's home, in a car but not driving or on holidays. Another issue found in this US study [24] was the higher proportion of calls to mobile phones resulting in ineligible respondents due to age (people excluded if less than 18 years of age), a lower response rate than calls to landline telephones and a higher refusal rate.

Furthermore, the selection of the respondent differs between mobile and landline telephones. The mobile telephone is usually individually owned and accessed by that one individual most of the time, compared to landline telephones that belong to a household which may be accessed by one or more people. Hence, consideration needs to be given when sampling strategies in terms of randomly selecting a single person to interview versus a number of eligible people in a household [16].

This study has highlighted the need to acquire a representative sampling frame and sampling methodology for household telephone (landline) surveys that minimises selection bias and is efficient in terms of administration and cost. With landline telephone numbers, the majority of the telephone numbers are listed in the EWP and the prefix of the telephone numbers are geographically based. Mobile telephones are the opposite; they are rarely listed (7.3% of mobile telephone users found in this study) and the number structure does not provide any details of geographical location, hence making it difficult to generate a sampling frame similar to current cost effective RDD methods. The large proportional difference in the EWP directory listing between landline and mobile telephone numbers would be mainly due to the options provided to the owners: owners of landline telephone need to pay to have their telephone numbers not listed in the EWP, and owners of mobile telephone need to pay to have their mobile telephone numbers in the EWP. Hence EWP samples are likely to continue to have a small proportion (6.9% in 2008) of mobile only households in the sampling frame. According to these results, if the option is to sample from the EWP, approximately 30% of the population will be excluded, particularly young people, those who have never married, those who reside in rural areas, people on lower income levels, the unemployed and students. In terms of health indicators, people in the normal weight range and current smokers could be under-represented.

Another emerging technology that has not been examined in this study is VoIP (Voice over Internet Protocol). In Australia, the impact of VoIP on sampling frames is not known. VoIP is seen as a cost effective system that utilises broadband data lines. Similar to mobile phones, the structure of VoIP telephone numbers (or also known as virtual number) are not geographically based and owners have the option of listing their VoIP telephone number in the EWP directory. More research is required on the uptake of VoIP including usage and impact on sampling frames.

The results of this study are potentially biased due to survey non-response. The response rates from these surveys (51.9% to 70.6%) could be considered moderately acceptable for a population survey of this kind. With increasingly inaccessible buildings (eg locked gates), busy lifestyles, and security and privacy concerns, an ongoing impact on response rates is expected, following patterns and trends interstate and overseas [25]. The unweighted age distribution had a higher proportion of older people and a lower proportion of younger people. This indicates the proportion of mobile only households could be under-estimated, and listings in the EWP over-estimated. Another limitation is the self-reported nature of this study. People might not want to divulge that they have a landline or mobile phone that is listed in the EWP because they want to avoid telephone calls from telemarketers or researchers [22] resulting in an under-estimation of telephone listings.

What does this mean for telephone (landline) surveys? Researchers need to be aware of the rapid changes in the telecommunication industry that potentially have an impact on collecting representative and reliable data on health-related issues using household telephone (landline) surveys. Studies like this are important because of the increasing need to monitor public health issues in a timely manner in an environment with limited and sometimes conflicting resources. Within these limits, there is a need to determine valid and reliable methods to verify the health estimates used for policy, planning of resources, and evaluation of health promotion interventions. Further research is needed in the area of mobile telephones such as how often the mobile is turned on, whether telephone calls are made more on the mobile or landline, and the likelihood of completing a health survey on a mobile telephone. Further Australian research is also required in terms of different weighting or post-survey adjustment strategies (eg raking) [26], improved sampling strategies [27] and the advantages and disadvantages of mixed mode surveying [28] (such as telephone, face-to-face, mail or internet), in order to improve the coverage of the sampling frame and minimise bias.

## Conclusion

Coverage of households with a telephone connected (landline) and the adequacy of the sampling frame(s) have been a concern for those involved in epidemiologically-sound telephone surveys. The rate of mobile only households in South Australia has been increasing and is following worldwide trends but has not reached the high levels seen internationally (12% to 52%). Presently, the impact of mobile telephones on current sampling frames (exclusion of mobile only households or non-listings in the White Pages directory) may be small in relation to the health estimates obtained using telephone surveys. However, researchers need to be aware that mobile only households have distinctly different characteristics compared to households with a landline connection and the increase in the number of mobile-only households is not uniform across all groups in the community. Listing in the White Pages directory is continuing to decrease and only a small proportion of mobile only households are listed. Researchers need to be aware of these telephone sampling issues when considering telephone surveys.

## Abbreviations

ABS: Australian Bureau of Statistics; BMI: Body Mass Index; CD: Collector Districts; EWP: Electronic White Pages; IRSD: Index of Relative Social Disadvantage; LA-RDD: List-assisted Random Digit Dialling; RDD: Random Digit Dialling; SPSS: Statistical Package for Social Sciences; VOIP: Voice over Internet Protocol

## Competing interests

The authors declare that they have no competing interests.

## Authors' contributions

EDG was responsible for the intellectual planning of the project, conception and design of the study, oversight of the Health Omnibus Survey, analysis of data, and interpretation of the data, and drafting of the article. AT was responsible for the intellectual planning of the project, oversight of the Health Omnibus Survey, interpretation of the data and revising of the paper for critical important intellectual content. All authors read and approved the final manuscript.

## Pre-publication history

The pre-publication history for this paper can be accessed here:

http://www.biomedcentral.com/1471-2288/10/77/prepub
